# A comparison of the release of phosphorus by a phytase enzyme in pigs fed diets deficient or adequate in phosphorus content

**DOI:** 10.1093/jas/skab001

**Published:** 2021-04-16

**Authors:** Kristin M Olsen, Stacie A Gould, John F Patience

**Affiliations:** 1 Department of Animal Science, Iowa State University, Ames, IA 50011-1178; 2 Iowa Pork Industry Center, Iowa State University, Ames, IA 50011-1178

**Keywords:** calcium balance, calcium retention, digestibility, phosphorus balance, phosphorus retention

## Abstract

Previous research indicated that phytase may release less phosphorus (P) from phytate when it is evaluated using diets with P levels above requirement as compared with diets below requirement. The objectives of this experiment were to further test the hypothesis that the P release values determined for phytase are higher when pigs are fed diets that are deficient (**DE**) in P compared with when they are fed diets that are adequate (**AD**) in P, and that phytase will increase the digestibility of dry matter (**DM**), gross energy (**GE**), nitrogen (N), and calcium (Ca) independent of dietary P status. Twenty-four barrows (body weight: 23.2 ± 1.8 kg) were randomly assigned to one of eight dietary treatments and housed in individual pens for 21 d and then moved to metabolism crates for 9 d, with the collection of urine and feces occurring on the final 5 d. A basal corn–soybean meal diet (P-AD) was formulated at 0.36% standardized total tract digestible (**STTD**) P and total calcium:STTD P (Ca:STTD P) of 2:1. A P-DE diet was also formulated to maintain a constant Ca:STTD P of 2:1 in both basal diets. Phytase was added to AD and DE diets at 350, 600, 1,000 phytase units (**FYT**)/kg. Pig was the experimental unit; diet (P-AD or P-DE), phytase level, and replicate were fixed effects. Orthogonal polynomial contrasts were used to test linear and quadratic effects of phytase within P-AD and P-DE diets. Phytase improved apparent total tract digestibility (**ATTD**) and STTD of P in both P-AD (linear *P* < 0.001) and P-DE diets (quadratic *P* < 0.001). Estimates for STTD P release were 0.07%, 0.09%, and 0.09% for 350, 600, and 1,000 phytase units (FYT)/kg in P-DE diets, and 0.02%, 0.03%, and 0.05% in P-AD diets, respectively. In P-DE diets, phytase improved absorption and retention of P and increased urinary excretion of P (quadratic *P* < 0.001). In P-AD diets, phytase improved absorption of P (linear *P* = 0.066), tended to improve retention (linear *P* = 0.066), and increased urinary excretion of P (quadratic *P* = 0.021). Phytase improved ATTD of Ca in P-DE diets (quadratic *P* = 0.002) but not in P-AD diets (*P* > 0.1). In conclusion, the release of P by phytase is lower in diets that are AD in P than those which are DE. Phytase increased the availability of Ca only in the diets DE in P. Finally, phytase increased the ATTD of DM and tended to increase the ATTD of energy, independent of dietary P status.

## Introduction

Phytate is an important component of ingredients of plant origin that are commonly used in swine diets. Most critically, the majority of P found in plants exists as salts of phytic acid, rendering it unavailable to the pig (Almeida et al., 2013). This increases the cost of feeding pigs, due to the need to supplement higher levels of inorganic P; it also has environmental implications due to the elevated concentration of P in manure. Since P is the third most expensive component of the diet, after energy and amino acids (**AA**), this is an issue of great concern to the global pig industry as well as consumers (Létourneau-Montminy et al., 2011). The discovery and development of the commercial application of the enzyme phytase, which hydrolyzes phytate to inorganic P and lower level inositol phosphates and free inositol, represent one of the great developments in swine nutrition in the past 50 yr. Phytase is now nearly universally employed in swine diets whenever additional available P is required.

In order to effectively and confidently formulate pig diets using phytases, their ability to release P must be quantified. One common approach to accomplish this is to employ growth titrations where a negative control diet containing levels of P below requirement is fed for 3 to 4 wk. To this negative control diet, one or more increments of phytase are included to provide up to, but slightly below, the expected P requirement. The improvement in growth performance and/or bone mineralization is then regressed against the level of phytase and compared with diets containing a known amount of available P or standardized total tract digestible (**STTD**) P, typically supplied by monocalcium phosphate (Kerr et al., 2010; Gourley et al., 2018). Feeding deficient (**DE**) levels of P allows growth performance or bone mineralization to be used to define the phytase release value (Jones et al., 2010). This approach has many advantages, including a rapid turnaround, relatively low cost, and statistical simplicity.

It is known that feeding diets DE in P may result in enhanced efficiency of absorption of dietary P; it may also stimulate bone resorption (Vipperman et al., 1974; Cromwell, 2005; Berndt and Kumar, 2009). Therefore, P release curves generated under conditions of P deficiency may not represent P release by phytase under normal physiological conditions. Olsen et al. (2018) reported that P release by phytase appeared to be lower when the test diet was adequate (**AD**) in P.

In addition to P, phytate is known to reduce the digestibility of other nutrients in the diet, such as cations like Ca, certain proteins, depending on their charge, and sources of energy such as starch (Humer et al., 2015). As expected, phytase has been shown to improve the availability of dietary components in addition to P, thus enhancing its value in the diet of the pig (Wensley et al., 2020).

Therefore, the primary objective of this study was to build on the results of our previous study (Olsen et al., 2018) to further test the hypothesis that the P release values determined for phytase are higher when pigs are fed diets that are DE in P compared with when they are fed diets that are AD in P. A secondary objective of the experiment was to test the hypothesis that phytase will increase the digestibility of dry matter (**DM**), gross energy (**GE**), N, and Ca in both P-DE and P-AD diets.

## Materials and Methods

All procedures employed in this experiment adhered to the principles for the ethical and humane use of animals for research as enunciated by the Guide for the Care and Use of Agricultural Animals in Research and Teaching (FASS, 2010) and were approved by the Iowa State University Animal Care and Use Committee (IACUC # 18-258).

### Animals, housing, and management

This experiment was conducted at the Iowa State University Swine Nutrition Farm (Ames, IA) from October 2018 to February 2019, using 72 crossbred barrows, the progeny of C22 or C29 sows × 337 terminal sires (PIC Inc., Hendersonville, TN) with an average initial body weight (**BW**) of 23.2 ± 1.8 kg. The experiment was conducted over three replicates of 24 animals each. Within replicate, pigs were assigned to one of eight dietary treatments using a completely randomized design. Pigs remained on their respective dietary treatments for the duration of the 30-d experiment. For the first 21 d of the experiment, pigs were housed in individual pens (1.8 × 2.7 m) equipped with 50% slatted concrete floors, a dry self-feeder, and a nipple waterer. For the first 19 d, pigs were fed ad libitum, and then they were limit fed at three times their daily estimated maintenance energy requirement (197 kcal of ME/kg BW^0.60^; NRC, 2012). The daily feed ration was given twice daily in equal meals at 0800 and 1600 hours. If a pig did not consume its entire meal after 1 h, orts were collected, weighed, and recorded for calculations of actual daily feed intake. On day 21, pigs were moved to metal metabolism crates, which facilitated the quantitative collection of daily urine output as well as uncontaminated grab samples of feces. Barrows were used in this experiment to enhance separate collection of urine and feces. They remained in these crates until the conclusion of the experiment on day 30. In order to limit luxury water consumption in limit-fed pigs (Fraser et al., 1993), water was provided at a 2.7:1 ratio to feed 1 h after each meal. This amount of water satisfies water requirements for pigs in a thermoneutral environment as described by Shaw et al. (2006).

### Experimental design and diets

Eight corn–soybean meal-based diets were formulated based on the NRC requirements for 25 to 50 kg BW growing pigs (NRC, 2012; Table 1). Prior to feed mixing, corn, soybean meal, monocalcium phosphate, limestone, vitamin and trace mineral premixes, salt, and synthetic AA were analyzed for Ca and P content to improve precision in the final diet composition. Two basal diets were formulated: one was a P-AD diet and another was P-DE. The AD diet contained 0.36% STTD P, considered to be above the pigs’ requirement, while DE contained 0.18% STTD P, considered to be well below the pigs’ requirement for P (NRC, 2012).

**Table 1.** Ingredient and nutrient composition of experimental diets (as-fed basis)

	Dietary treatment	
	AD^1^	DE^2^
Ingredient, %		
Corn	71.65	73.15
Soybean meal (47.5% CP)	23.64	23.69
Soybean oil	0.89	0.89
Monocalcium phosphate	1.36	0.32
Limestone	0.80	0.31
l-Lys HCl	0.29	0.28
dl-Methionine	0.06	0.06
l-Threonine	0.06	0.06
Vitamin premix^3^	0.25	0.25
Trace mineral premix^4^	0.20	0.20
Titanium dioxide	0.40	0.40
Sodium chloride	0.40	0.40
Calculated nutrients, %		
STTD phosphorus	0.36	0.18
Total phosphorus	0.57	0.37
Total calcium	0.76	0.38
Total Ca:STTD P	2.10	2.10
Net energy, Mcal/kg	2.48	2.52
Standardized ileal digestible (SID) lysine	0.98	0.98
SID methionine	0.30	0.30
SID total sulfur AA	0.55	0.56
SID threonine	0.59	0.59
SID tryptophan	0.18	0.18

^1^There were four diets containing 0.36% STTD P (AD). Phytase was added at the expense of corn in the following amounts: 0, 350, 600, and 1,000 phytase units (FYT) phytase/kg (Ronozyme HiPhos 2500GT, DSM Nutritional Products Inc., Parsippany, NJ, USA; treatments AD, AD350, AD600, and AD1000).

^2^There were four diets containing 0.18% STTD P (DE). Phytase was added at the expense of corn in the following amounts: 0, 350, 600, and 1,000 FYT phytase/kg (Ronozyme HiPhos 2500GT, DSM Nutritional Products Inc., Parsippany, NJ, USA; treatments DE, DE350, DE600, and DE1000).

^3^Premix provided per kg of complete diet: 7,656 IU vitamin A, 875 IU vitamin D3, 63 IU vitamin E, 4 mg vitamin K, 14 mg riboflavin, 70 mg niacin, 34 mg pantothenic acid, and 63 mg vitamin B_12._

^4^Premix provided per kg of complete diet: 220 mg Fe (ferrous sulfate), 220 mg Zn (zinc sulfate), 52 mg Mn (manganese sulfate), 22 mg Cu (copper sulfate), 0.4 mg I (calcium iodate), and 0.3 mg Se (sodium selenite).

To achieve the objective of comparing P release by phytase in a P-AD vs. a P-DE diet, three levels of a 6-phytase (EC 3.1.3.26) derived from *Citrobacter braakii* and expressed in *Aspergillus oryzae* (Ronozyme HiPhos 2500GT, DSM Nutritional Products Inc., Parsippany, NJ, USA) were added at the expense of corn to AD and DE in the following amounts: 350, 600, and 1,000 phytase units (**FYT**)/kg. The phytase source was pre-analyzed for phytase activity, using a modification of AOAC (2000) method 2000.12 (Engelen et al., 1994, 2001) and was determined to contain 2,700 FYT/g. This analyzed activity was used to calculate the amount of phytase required to achieve the desired phytase activity in the final mixed diets.

Both basal diets were formulated to contain a 2:1 Ca:STTD P. This ratio, based on the P content of the basal diet, was employed in both the AD and DE series of diets to avoid confounding the experiment with varying Ca:STTD P ratios, which are known to impact the efficacy of phytase (Beaulieu et al., 2007; Selle and Ravindran, 2008; Wu et al., 2018). The level of dietary Ca was kept constant while phytase increased in the diets; increasing the level of dietary Ca as phytase supplementation increased would make it impossible to distinguish between the response to phytase and any possible response to increasing Ca or limestone; this is particularly true when Ca and P balance and retention are being studied (Tamin et al., 2002).

Titanium dioxide (TiO_2_) was added at 0.4% to all the diets as an indigestible marker. To achieve precision in diet mixing, ingredients with low inclusion rates (vitamin and trace mineral premixes, monocalcium phosphate, limestone, synthetic AA, TiO_2_, and phytase) were weighed on an analytical scale and premixed in a small mixer (approximately 57 liters capacity, Hobart Corporation, Troy, OH) before being added with the bulk ingredients to the main batch mixer. Scales were validated with standard check weights prior to mixing.

### Sample and data collection

Two 1 kg samples of feed were collected during feed mixing: one sample was taken directly from the mixer as feed was dispensed, and the other was taken using a feed probe from each of the barrels in which diets were stored after mixing. These samples were pooled and homogenized and stored at −20 °C until further analysis. Total urine output and fecal grab samples were collected twice a day during the last 5 d of the experiment, (day 26 to 30 inclusive). Urine was collected in acid-washed containers preloaded with 20 mL of HCl to minimize nitrogen losses through ammonia volatilization and to control microbial breakdown of urine contents. Twice a day, urine was harvested, filtered through glass wool, subsampled, and stored in acid-washed plastic containers at −20 °C until further analysis.

### Chemical analysis

At the conclusion of each replicate, urine and fecal samples were thawed at room temperature, pooled for each pig, homogenized, and subsampled for subsequent chemical analysis. Urine subsamples were re-stored at −20 °C; at the time of analyses, urine subsamples were thawed, mixed to ensure sample homogeneity, and filtered through Whatman 41 filter paper (GE Healthcare Life Sciences, Chicago, IL, USA).

Fecal samples were dried in a convection oven at 75 °C until a constant weight was achieved. Dried fecal samples and feed samples were ground through a 1-mm screen and stored in desiccators. Diet and fecal samples were analyzed for DM (method 990.03; AOAC, 2007), TiO_2_ (Leone, 1973), GE, and N. GE was determined using an isoperibolic bomb calorimeter (Parr 6200 calorimeter; Parr Instruments Co., Moline, IL); benzoic acid (6,318 cal GE/kg; Parr Instruments Co.) was used as the standard for calibration and was determined to contain 6,319 ± 12 cal GE/g. Total N was analyzed using the combustion method (method 990.03; AOAC, 2007) with a TruMac apparatus (Leco Corporation, St. Joseph, MI). Ethylenediaminetetraacetic acid (9.56% N) was used for calibration and was determined to contain 9.61 ± 0.12% N. Crude protein was calculated as N × 6.25. A maximum of 1% coefficient of variation (CV) between duplicates was required for GE, N, and DM. A maximum CV of 5% was required for TiO_2_. If the CV between duplicates of a sample exceeded these maximums, the sample was rerun in duplicate. Diet, fecal, and urine samples were analyzed for P and Ca by inductively coupled plasma optical emission spectrometry at a commercial laboratory (Eurofins Nutrition Analysis Center, Des Moines, IA). Diets were also analyzed for phytase activity using a slight modification of AOAC (2000) method 2002.12 (Engelen et al., 1994, 2001) and phytate-bound P content using near-infrared spectroscopy (Latta and Eskin, 1980).

### Calculations

To determine the daily mean values for total DM intake and urine output of P and Ca, 5 d totals were recorded and divided by day within the collection period (g/d). Apparent total tract digestibility (**ATTD**) of nutrients and fecal output were calculated according to Oresanya et al. (2008). STTD of P was calculated as described in NRC (2012), assuming basal endogenous losses of P to be 190 mg/kg dry matter intake (DMI). Apparent mineral absorption and net retention (referred to hereafter as absorption and retention) were calculated on a DM basis (g/d) as:

$$\begin{array}{l} Apparent\;absorption\;=\;mineral\;intake-mineral\;in\;feces \end{array}$$ (1)

$$\begin{array}{l} Net\;retention\;=\;mineral\;intake-mineral\;in\;feces-mineral\;in\;urine \end{array}$$ (2)

Retention of P, as a percentage of P intake and of P absorbed, was also calculated as follows:

$$\begin{array}{l} Retention,\;\%\;of\;intake\;=\;retention\;/\;P\;intake\;\times \;100 \end{array}$$ (3)

$$\begin{array}{l} Retention,\;\%\;of\;absorbed\;=\;retention\;/\;absorbed\;P\;\times \;100 \end{array}$$ (4)

The STTD P release value for each phytase level was estimated by multiplying the total P content of the diet by the improvement in STTD of P based on regression equations (Table 3).

### Statistical analysis

Outliers, defined as studentized residuals greater than 3 standard deviations from zero, were identified and removed. The normality of the residuals was verified using the Shapiro–Wilk’s test. Residual plots were examined to confirm that the assumptions of equal variances were met. Data were analyzed in a 2 × 4 factorial model including the fixed effects of diet (P-AD or P-DE), phytase level, replicate, and all possible interactions (diet × phytase level, diet × replicate, phytase level × replicate, and diet × phytase level × replicate). Except for the interaction between diet and phytase level, which was included in all analyses, interaction terms were removed from the model when *P* > 0.30. Orthogonal polynomial contrasts were performed within the four P-AD diets (AD, AD350, AD600, and AD1000) and the four P-DE diets (DE, DE350, DE600, and DE1000) to test the linear and quadratic effects of phytase level on selected response variables (Table 2). Orthogonal polynomial contrasts were also performed to test the linear and quadratic effects of phytase level across both diets (P-AD and P-DE) where the diet × phytase interaction was not significant (ATTD DM, ATTD GE, and ATTD crude protein [CP]). Coefficients for unequally spaced phytase levels were obtained with the interactive matrix language procedure (PROC IML) of SAS. Differences were considered significant if *P* < 0.05, and trends if 0.05 < *P* < 0.10. SAS 9.4 (SAS Inst. Inc., Cary, NC) was used for all analyses with GLM and UNIVARIATE procedures used for statistical analyses and outlier identification, respectively.

**Table 2.** Analyzed nutrient composition of experimental diets (as-fed basis)

	Dietary treatment^1^							
Nutrient, %	AD	AD350	AD600	AD1000	DE	DE350	DE600	DE1000
DM	88.4	88.4	88.0	88.2	88.1	87.9	88.1	88.2
GE, Mcal/kg	4.06	4.04	4.01	4.06	4.13	4.15	4.15	4.09
Crude protein	15.8	15.9	16.2	15.7	16.1	16.2	16.3	15.7
Total calcium	0.71	0.68	0.67	0.63	0.34	0.33	0.35	0.34
Total phosphorus	0.59	0.58	0.60	0.56	0.39	0.40	0.39	0.39
Phytate-bound phosphorus	0.27	0.26	0.27	0.26	0.24	0.24	0.24	0.24
Phytase activity, FYT/kg	228	440	552	946	98	319	628	1,069

^1^There were a total of eight dietary treatments. Diets 1 to 4 were P-AD diets: (0.36% STTD P) with 0 (AD), 350 (AD350), 600 (AD600), and 1,000 (AD1000) phytase units (FYT)/kg (Ronozyme HiPhos 2500GT, DSM Nutritional Products Inc., Parsippany, NJ, USA), respectively. Diets 5 to 8 were P-DE diets (0.18% STTD P) with 0 (DE), 350 (DE350), 600 (DE600), and 1,000 (DE1000) FYT/kg. Phytase was added at the expense of corn.

## Results

### Effect of phytase on P balance

An interaction was observed between the P-DE and the P-AD series of diets (Table 3; *P* < 0.001) for all aspects of P metabolism with the exception of urine excretion and P retention as a proportion of P absorbed. Pigs on either diet series experienced increased urinary P losses as phytase increased (*P* < 0.001) and reduced P retention as a portion of absorbed P (*P* < 0.001).

**Table 3.** Effect of dietary treatment of digestibility and balance of P in growing pigs

	Dietary treatment^1^									*P*-value^2^				
	AD	AD350	AD600	AD1000	DE	DE350	DE600	DE1000	SEM	Diet	FYT	Diet * FYT	AD Con^2,3^	DE Con^2,3^
P intake, g/d	9.7	9.6	9.9	9.5	6.2	6.5	6.1	6.3	—	—	—	—	—	—
ATTD, %	46.3	52.3	52.2	55.8	33.8	53.2	57.2	58.7	1.2	0.266	<0.001	<0.001	L, <0.001	Q, <0.001
STTD^4^, %	50.5	56.3	56.3	60.1	39.9	59.2	63.1	64.6	1.3	0.303	<0.001	<0.001	L, <0.001	Q, <0.001
Absorbed, g/d	4.5	5.0	5.2	5.3	2.1	3.5	3.5	3.7	0.1	<0.001	<0.001	0.010	L, <0.001	Q, <0.001
Retained, g/d	3.8	4.2	4.1	4.3	2.0	3.1	3.1	3.2	0.1	<0.001	<0.001	0.020	L, 0.066	Q, <0.001
Urine, g/d	0.7	0.8	1.1	1.0	0.1	0.4	0.4	0.5	0.1	<0.001	<0.001	0.133	Q, 0.021	Q, <0.001
Fecal, g/d	5.2	4.6	4.8	4.2	4.1	3.0	2.6	2.6	0.1	<0.001	<0.001	<0.001	L, <0.001	Q, <0.001
Total excreted, g/d	5.9	5.4	5.9	5.2	4.2	3.4	3.0	3.1	0.1	<0.001	<0.001	<0.001	L, <0.001	Q, <0.001
Retention, % ab^5^	84.6	82.9	78.7	80.5	94.9	88.8	87.5	86.8	1.2	<0.001	<0.001	0.234	L, 0.006	Q, 0.020
Retention, % in^6^	39.1	43.5	41.0	44.8	32.1	47.0	49.9	50.9	1.3	<0.001	<0.001	<0.001	L, 0.012	Q, <0.001

^1^There were a total of eight dietary treatments. Diets 1 to 4 were P-AD diets: (0.36% STTD P) with 0 (AD), 350 (AD350), 600 (AD600), and 1,000 (AD1000) phytase units (FYT)/kg (Ronozyme HiPhos 2500GT, DSM Nutritional Products Inc., Parsippany, NJ, USA), respectively. Diets 5 to 8 were P-DE diets (0.18% STTD P) with 0 (DE), 350 (DE350), 600 (DE600), and 1,000 (DE1000) FYT/kg. Phytase was added at the expense of corn.

^2^Diet and FYT represent *P*-values for main effects of dietary P level (P-AD vs. P-DE) and phytase level, respectively. Diet * FYT represents *P-*value for interaction between dietary P level and phytase level. AD Con and DE Con represent *P*-values (linear [L] and quadratic [Q]) for orthogonal polynomial contrasts performed to test the effect of phytase level within the P-AD and P-DE diets, respectively.

^3^Resulting regression equations for selected traits are as follows: In P-AD diets ATTD of P, % = 47.35 + 0.0089 (x); STTD of P, % = 51.40 + 0.0089 (x); and Urine P, g/d = 0.69 + 0.00089(x) – 0.00000051(x^2^). In P-DE diets ATTD of P, % = 34.18 + 0.064 (x) – 0.000041 (x^2^); STTD of P, % = 40.29 + 0.064 (x) – 0.00004(x^2^); Urine P, g/d = 0.11 + 0.00096(x) – 0.00000061(x^2^); and Retained P, g/d = 2.02 + 0.00096 (x) – 0.000002(x^2^). x = phytase units (FYT/kg).

^4^STTD P was calculated assuming 190 mg endogenous P losses/kg DMI based on NRC (2012).

^5^Retention of P as a percentage of absorbed P.

^6^Retention of P as a percentage of P intake.

Otherwise, in the P-AD diets, the ATTD and STTD of P increased linearly with phytase levels (*P* < 0.001). The greatest digestibility of P was observed in diets AD1000. The quantity of absorbed P increased linearly with phytase (*P* < 0.001), while retention of P only tended to increase (quadratic *P* = 0.066). The quantity of fecal losses of P declined with increasing phytase (linear *P* < 0.001). Thus, P retention, expressed as a percentage of daily intake, increased in a linear fashion (*P* = 0.012). These results were largely as expected.

In the P-DE diets, designated as DE, the ATTD and STTD of P increased with increasing levels of phytase in a quadratic fashion, with the biggest increase occurring between 0 and 350 FYT and a less pronounced relative increase in the 600 and 1,000 FYT treatments (*P* < 0.001). The ATTD and STTD of P were greater than those in the AD series of diets, except for the non-phytase-containing diets; in these diets, the DE had lower ATTD and STTD of P than the AD (Diet × FYT; *P* < 0.001). Absorbed and retained P increased in a quadratic pattern with phytase level (*P* < 0.001), again with the biggest increase from 0 to 350 FYT and appearing to plateau in the diets with 350, 600, or 1,000 FYT. Fecal losses of P as well as total excreted P all declined with increased phytase (quadratic *P* < 0.001). Retained P as a portion of total intake increased with increasing levels of phytase (*P* < 0.001).

### Effect of phytase on Ca balance

An interaction in Ca metabolism was also observed between the P-DE and the P-AD series of diets (Table 4; *P* < 0.015) with the exception of fecal and total excretion as well as retention as a proportion of absorbed Ca.

**Table 4.** Effect of dietary treatment on digestibility and balance of Ca in growing pigs

	Dietary treatment^1^									*P*-value				
	AD	AD350	AD600	AD1000	DE	DE350	DE600	DE1000	SEM	Diet	FYT	Diet * FYT	AD Con^2,3^	DE Con^2,3^
Ca intake, g/d	12.0	11.3	11.4	10.9	5.5	5.7	5.6	5.7	—	—	—	—	—	—
ATTD, %	54.8	59.3	54.9	57.1	63.1	73.3	74.6	74.9	1.5	<0.001	<0.001	0.003	NS	Q, 0.002
Absorbed, g/d	6.5	6.7	6.3	6.2	3.5	4.2	4.2	4.3	0.2	<0.001	0.157	0.013	NS	L, 0.003
Retained, g/d	6.2	6.6	6.1	6.0	3.0	4.1	4.1	4.3	0.2	<0.001	0.002	0.001	NS	Q, 0.01
Urine, g/d	0.31	0.08	0.19	0.16	0.55	0.04	0.04	0.03	0.02	<0.001	<0.001	<0.001	Q, <0.001	Q, <0.001
Fecal, g/d	5.4	4.6	5.11	4.7	2.1	1.5	1.4	1.4	0.2	<0.001	<0.001	0.225	L, 0.007	L, 0.006*
Total excreted, g/d	5.7	4.7	5.3	4.9	2.5	1.6	1.5	1.5	0.2	<0.001	<0.001	0.100	Q, 0.042	Q, 0.002
Retention, % ab^4^	84.6	82.9	78.7	80.5	94.9	88.8	87.5	86.8	1.2	<0.001	<0.001	0.234	L, 0.006	Q, 0.020
Retention, % in^5^	39.1	43.5	41.0	44.8	32.1	47.0	49.9	50.9	1.3	<0.001	<0.001	<0.001	L, 0.012	Q, <0.001

^1^There were a total of eight dietary treatments. Diets 1 to 4 were P-AD diets: (0.36 % STTD P) with 0 (AD), 350 (AD350), 600 (AD600), and 1,000 (AD1000) phytase units (FYT)/kg (Ronozyme HiPhos 2500GT, DSM Nutritional Products Inc., Parsippany, NJ, USA), respectively. Diets 5 to 8 were P-DE diets (0.18% STTD P) with 0 (DE), 350 (DE350), 600 (DE600), and 1,000 (DE1000) FYT/kg. Phytase was added at the expense of corn.

^2^Diet and FYT represent *P*-values for main effects of dietary P-level (P-AD vs. P-DE) and phytase level, respectively. Diet * FYT represents *P-*value for interaction between dietary P-level and phytase level. AD Con and DE Con represent *P*-values (linear [L] and quadratic [Q]) for orthogonal polynomial contrasts performed to test the effect of phytase level within the P-AD and P-DE diets, respectively.

^3^Regression equations for selected traits are as follows: In P-DE diets: ATTD Ca, % = 63.34 + 0.033(x) – 0.000022(x^2^); Retained Ca, g/d = 3.08 + 0.0032(x) – 0.000002(x^2^), where x = phytase units (FYT/kg).

^4^Retention of Ca as a percentage of absorbed Ca.

^5^Retention of Ca as a percentage of Ca intake.

*Quadratic *P*-value 0.0645.

In the P-AD diets, phytase had no effect on the ATTD of Ca as well as daily absorption and daily retention of Ca. Retention of Ca as a percent of intake increased with increasing phytase (*P* < 0.001). This seemingly inconsistent finding, given the previously mentioned absence of phytase effect, is due to the combination of differences in Ca intake (based on assay rather than on formulated values), urinary output, and fecal output, which alone were not different.

In the P-DE diets, the ATTD of Ca increased in a quadratic fashion with increasing phytase (*P* = 0.002). As a result, the daily quantity of Ca absorbed and retained increased in a linear manner (*P* = 0.003). Urinary excretion of Ca declined in a quadratic fashion reaching a constant level in the diets containing 350, 600, or 1,000 FYT (*P* < 0.001). The overall retention of Ca increased within increasing phytase in a quadratic manner with the biggest increase occurring between 0 and 350 FYT (*P* < 0.001).

### Results of regression analysis and estimation of P and Ca release by phytase

The improvement in digestibility (STTD of P) for a given phytase level as indicated by the corresponding regression equation (Table 3) was multiplied by the total P content of the diet to estimate STTD P release. In P-AD diets, phytase was estimated to release 0.02%, 0.03%, and 0.05% STTD P for 350, 600, and 1,000 FYT/kg, respectively. In P-DE diets, phytase was estimated to release 0.07%, 0.09%, and 0.09% STTD P for these same phytase levels. There was no significant improvement of ATTD of Ca in P-AD diets, but in P-DE diets, phytase was estimated to release 0.03%, 0.04%, and 0.04% ATTD Ca for 350, 600, and 1,000 FYT/kg, respectively (Table 4).

### Effect of phytase on ATTD of energy and other nutrients

There were no interactions between diet and phytase level for ATTD of DM, GE, or CP (Table 5; *P* > 0.1), and there was no effect of dietary level of P on these parameters (*P* > 0.1). There was a significant effect of phytase level on ATTD of DM (*P* = 0.032), with a slight improvement in diets with 350 or 600 FYT/kg (quadratic *P* = 0.004). There was also a tendency for increased GE digestibility in diets with 350 or 600 FYT/kg (*P* = 0.059; quadratic *P* = 0.018). Crude protein digestibility tended to increase with phytase; the highest CP digestibility was observed in the diets containing 1,000 FYT/kg (*P* = 0.098, quadratic *P* = 0.027).

**Table 5.** Effect of phytase and dietary P-level on ATTD of DM, GE, and CP

	Dietary treatments^1^						*P*-value^2^			
	0	350	600	1,000	AD	DE	SEM	FYT	Diet	FYT Con^3^
DM, %	86.3	87.1	87.3	86.3	86.7	86.8	0.3	0.032	0.711	Q, 0.004
GE, %	85.5	86.0	86.2	85.0	85.8	85.5	0.4	0.059	0.424	Q, 0.018
CP,%	83.2	83.9	84.7	85.5	83.3	83.8	0.2	0.098	0.432	Q, 0.027

^1^There were a total of eight dietary treatments. Diets 1 to 4 were P-AD diets: (0.36% STTD P) with 0 (AD), 350 (AD350), 600 (AD600), and 1,000 (AD1000) phytase units (FYT)/kg (Ronozyme HiPhos 2500GT, DSM Nutritional Products Inc., Parsippany, NJ, USA), respectively. Diets 5 to 8 were P-DE diets (0.18% STTD P) with 0 (DE), 350 (DE350), 600 (DE600), and 1,000 (DE1000) FYT/kg. Phytase was added at the expense of corn.

^2^No interaction between diet and phytase level, so main effects are presented.

^3^FYT Con represents *P*-values (quadratic [Q]) for orthogonal polynomial contrasts performed to test the effect of phytase level.

## Discussion

The STTD P release values for phytase were lower in P-AD diets than in P-DE diets. For example, when 350 FYT/kg was added to the P-AD diet, there was about a 12% increase in STTD of P, but in the P-DE diet, phytase yielded a 48% increase. Although the response to phytase was quadratic in the P-DE diets, 1,000 FYT/kg still released 0.04% more STTD P than in the P-AD diets. Thus, this experiment supported our first hypothesis that the apparent release of P by phytase is greater in P-DE than in P-AD diets. In this regard, it agrees with the results previously reported by Olsen et al. (2018) and confirmed more recently by Tsai et al. (2020). The outcome of lower P digestibility in the DE vs. AD control diet was also expected; when a larger proportion of the P in the diet is from a plant source (corn and soybean meal) rather than an inorganic source (monocalcium phosphate), as was the case in the DE vs. the AD diet, a smaller proportion of the dietary P is digestible and thus results in a lower percentage of ATTD or STTD P. This result was similar to the observations reported in Olsen et al. (2018). The responses of P metabolism to phytase were largely as expected, except for the quadratic nature of the response to phytase; linear responses were expected, since the highest level of phytase, at 1,000 FYT/kg, would not be considered very high. However, a quadratic response to phytase is not at all unusual (Jones et al., 2010).

The different outcomes between diets AD or DE in P could be due to several factors. It is well known that P homeostasis is a carefully regulated process involving a complex set of regulatory systems, including the vitamin D endocrine system, parathyroid hormone, and phosphatonins affecting gastrointestinal absorption and bone metabolism among other systems (Berndt and Kumar, 2009). Low dietary P intake, such as would have been experienced on the P-DE diets, results in a proportionate increase in absorption of P by the gut. Therefore, it is possible that P released by phytase could have been absorbed with greater efficiency in the P-DE diets than in those which were P-AD. Another possibility is the powerful negative feedback of inorganic P on phytase function; in other words, as phytase releases more P, accumulation of P in the intestinal digesta will inhibit enzyme function, a classical feedback loop (Lei and Stahl, 2000).

Data from our previous experiment demonstrated that, as phytase made P more available, pigs were able to continue to retain P well after the requirement of P for growth was met (NRC, 2012; Olsen et al., 2018). In the current study, there was only a tendency for retained P to increase with phytase in P-AD diets. In our previous study, absorbed P reached 5.8 g/d, and pigs were able to retain as much as 5.0 g of P/d. In the current experiment, absorption of P only increased to 5.3 g/d; thus, retained P did not increase as much either. The slight differences in P intake may also partially explain the lack of differences in retained P. Data from Gutierrez et al. (2015) indicate that the intake of STTD P needed to reach maximum bone P accretion is approximately 8.8 g/d. All diets in this experiment would have provided less than 8.8 g/d of STTD P intake. Therefore, pigs should have been able to continue retaining P if absorbed P had increased more dramatically with phytase. It has also been demonstrated that pigs will continue to retain P as long as Ca and P are balanced in the diet (Létourneau-Montminy et al., 2012). In the present experiment, dietary Ca levels were kept constant as phytase was added to the diets. Absorbed Ca did not increase significantly with phytase in the P-AD diets, meaning the effective Ca:P ratio would have decreased as absorbed P increased with phytase. Furthermore, the analyzed Ca content was slightly below expected. Both of these factors could have limited the pigs’ ability to retain P in bone.

For these reasons, our data are not able to fully confirm our previous findings that P retention increases at levels well above the requirement for growth. However, previous data indicate that the requirement of P for bone development is higher than the requirement for growth. Taking this in combination with the finding that phytase may release less P than expected if diets are formulated at the P requirement the implications for developing gilts are still valid. If developing gilts are fed P levels based on the requirement for growth, and phytase P release values may be lower than expected, we may be underfeeding P relative to the quantity required for optimal bone development and, ultimately, sow longevity. Considering lameness remains one of the most prevalent reasons for females to leave breeding herds, this is a question worthy of further investigation. In the short term, feeding developing gilts P levels well above their requirement for growth, a strategy already utilized by many in the commercial industry, is supported.

Phytase improved the ATTD of Ca in the P-DE diets but not in the P-AD diets. In P-DE diets, the response was quadratic with very little additional improvement in Ca digestibility beyond 350 FYT/kg. This is contradictory to our previous study, where phytase improved ATTD of Ca in P-AD diets in a linear fashion up to 800 FTU phytase/kg. González-Vega et al. (2015) also reported improvements in Ca digestibility in diets that were AD in P and Ca. The calculated Ca release in the P-DE diets was similar to previous estimates of phytase Ca release (González-Vega et al., 2015; Olsen et al., 2018), although they are lower than the manufacturer’s suggested Ca release of 0.089%, 0.129%, and 0.144% for 350, 600, and 1,000 FYT, respectively. Almeida et al. (2013) evaluated the same phytase as in the current experiment and reported similar improvements in ATTD Ca when phytase was added to P-DE diets. Phytase’s ability to improve Ca digestibility has been well documented (Guggenbuhl et al., 2012; Torrallardona et al., 2012; Rutherfurd et al., 2014).

 Interestingly, urine Ca reached very low levels when phytase was added to the P-DE diets. The higher amount of urinary Ca excretion in DE is consistent with hypercalciuria that often occurs when pigs are fed diets limiting in P. However, the low level of urine Ca in diets DE350, DE600, and DE1000 was surprising and could possibly be due to the lower increase in Ca digestibility relative to P digestibility. Again, this may have caused a low effective Ca:P ratio, resulting in pigs retaining most of the Ca absorbed but not all of the P absorbed. This could also explain why urinary P levels in the P-DE diets with phytase were above basal levels (Gutierrez et al., 2015).

Phytate can also bind other nutrients, including AA, starch, and fatty acids. Many studies have reported improvements in the digestibility of these nutrients when phytase is used, though results have been inconsistent (Selle et al., 2000; Adeola and Cowieson, 2011). Only very modest improvement in the ATTD of nutrients other than P and Ca was observed in this study, with slight improvements in DM, GE, and CP digestibility due to phytase. The lack of diet × phytase interaction indicates that there was no difference in the ability of phytase to improve the digestibility of DM, GE, or CP in P-AD diets vs. P-DE diets. Some studies have suggested that quite high levels of phytase are required to see effects on the digestibility of nutrients other than P and Ca (Zeng et al., 2016; Acosta and Patience, 2019).

The most difficult decision in the design of this experiment was the selection of the Ca and P levels in both the P-AD and the P-DE basal diets. Considering all of the options, and the impact of each on the experimental outcome, a constant Ca:STTD P ratio across both basal diets—AD and DE—was selected. Different Ca:STTD P ratios in the P-AD and in the P-DE diets would have confounded the experiment and begged the question as to whether any observed responses were due to a different P level—the primary focus of the experiment—or due to a different Ca:STTD P. This was, therefore, considered an unacceptable option. Our next decision was to select the ratio of Ca:STTD P. It is well known that a wide ratio will negatively impact phytase function, especially in diets near or below the pig’s requirement for P (Qian et al., 1996; Liu et al., 1998; Beaulieu et al., 2007; Selle and Ravindran, 2008; Dersjant-Li et al., 2015; Wu et al., 2018). A Ca:STTD P ratio of 2.1:1 was selected based on the results of other research and a desire to permit as high a level of Ca as possible in the P-DE diet. The final decision in the design of the experimental diets was to choose the P levels in the basal diets. A value about 12% above requirement was chosen for the P-AD diet, recognizing that the pigs would be limit fed, thus lowering daily P intake, and accommodating variation in formulated vs. actual levels in the final mixed diets. The resulting daily P intake remained above requirement (NRC, 2012), so this target was met. The level of P in the P-DE diet was chosen based on previous experience (Olsen et al., 2018; Acosta and Patience, 2019) to achieve sufficient impairment of P intake to achieve experimental objectives while avoiding pressure placed on animal well-being due to impaired skeletal development. Previously published researches seeking to achieve similar objectives were also consulted (Jones et al., 2010; Kerr et al., 2010; Kühn and Partanen, 2012; Taylor et al., 2016; Vier et al., 2019). Despite our best efforts, no single experiment can answer all questions, and we cannot declare that a different approach to the Ca level would not have resulted in a different outcome. Our suggestion would be to undertake an experiment to compare the Ca level in a study of this nature—a metabolism study—to determine if a different conclusion would be reached about the P release achieved by phytase. Certainly, there have been many studies on the topic in growth trials investigating phytase efficacy and the Ca:P ratio; examples include Qian et al. (1996), Liu et al. (1998), and Wu et al. (2018). However, none of the studies have incorporated a sufficient number of Ca:P ratios to titrate out the optimum ratio for both metabolism and growth.

In conclusion, the release of P by phytase is lower in diets that are AD in P than those which are DE. This is consistent with the findings of Olsen et al. (2018) and, more recently, Tsai et al. (2020). Interestingly, the increase in the availability of Ca through the use of phytase was only observed in diets that were DE in P; this is not consistent with other studies and, therefore, needs additional investigation. Finally, phytase increased the ATTD of DM and tended to increase the ATTD of energy, independent of dietary P status.

## Abbreviations

AA: amino acids
AD: diet adequate in phosphorus content
ATTD: apparent total tract digestible/digestibility
BW: body weight
DE: diet deficient in phosphorus content
DM: dry matter
DMI: dry matter intake
FYT: phytase units
GE: gross energy
SID: standardized ileal digestible
STTD: standardized total tract digestible/digestibility
